# Supplementary material for: Smoking cessation and prognosis during long-term follow-up after stroke, TIA, and acute coronary syndrome—results from the randomized controlled NAILED trial
Source: PLoS One. 2024 Nov 11;19(11):e0311955. doi: 10.1371/journal.pone.0311955 (PMC11554128; doi:10.1371/journal.pone.0311955)
Supplement: S1 Table — (DOCX) [file pone.0311955.s002.docx]

**S1 Table.** **Population characteristics associated with smoking status at 1 month and at the last follow-up**.

|  | **1 month** | | | **Last follow-up** | | |
| --- | --- | --- | --- | --- | --- | --- |
|  | Smoking | Non-smoking | p-value | Smoking | Non-smoking | p-value |
| N | 142 | 179 |  | 150 | 171 |  |
| Age, years, mean (SD) | 65.0 (9.8) | 62.8 (10.1) | 0.045 | 64.9 (9.7) | 62.8 (10.2) | 0.050 |
| Index event: ACS, N (%) | 69 (48.6) | 126 (70.4) | <0.001 | 82 (54.7) | 113 (66.1) | 0.037 |
| *STEMI* | 26 | 51 |  | 32 | 45 |  |
| *NSTEMI* | 40 | 68 |  | 46 | 62 |  |
| *UA* | 3 | 7 |  | 4 | 6 |  |
| Index event: TIA, N (%) | 31 (21.8) | 10 (5.6) | <0.001 | 25 (16.7) | 16 (9.4) | NS |
| Index event: stroke, N (%) | 42 (29.6) | 43 (24.0) | NS | 43 (28.7) | 42 (24.6) | NS |
| Previous vascular events, N (%) | 36 (25.4) | 30 (16.8) | 0.059 | 31 (20.7) | 35 (20.5) | NS |
| Antihypertensive drug(s), N (%) | 113 (79.6) | 158 (88.3) | 0.033 | 125 (83.3) | 146 (85.4) | NS |
| *1 drug* | 35 (24.6) | 31 (17.3) | NS | 37 (24.7) | 29 (17.0) | NS |
| *2 drugs* | 45 (31.7) | 77 (43.0) | 0.038 | 54 (36.0) | 68 (39.8) | NS |
| *≥3 drugs* | 33 (23.2) | 50 (27.9) | NS | 34 (22.7) | 49 (28.7) | NS |
| DAPT | 74 (52.1) | 114 (63.7) | 0.037 | 85 (56.7) | 103 (60.2) | NS |

No associations were found for the remaining population characteristics listed in Table 1. In addition, no association was found between smoking status and the mean BP/LDL-C level during follow-up. Previous vascular events was a composite of previous ischemic heart disease (previous acute myocardial infarction, percutaneous coronary intervention or coronary artery by-pass grafting), previous stroke or TIA, or previous peripheral artery disease . Drug treatment variables refers to treatment at discharge. SD, standard deviation; ACS, acute coronary syndrome; STEMI, ST elevation myocardial infarction; NSTEMI, non–ST elevation myocardial infarction; UA, unstable angina; TIA, transient ischemic attack; DAPT, dual antiplatelet therapy.
