# Supplementary material for: Smoking cessation and prognosis during long-term follow-up after stroke, TIA, and acute coronary syndrome—results from the randomized controlled NAILED trial
Source: PLoS One. 2024 Nov 11;19(11):e0311955. doi: 10.1371/journal.pone.0311955 (PMC11554128; doi:10.1371/journal.pone.0311955)
Supplement: S1 File — (DOCX) [file pone.0311955.s003.docx]

S1 File

**Definition of outcome events**

With minor modifications, the definitions found in this document are based on the Standardized Data Collection for Cardiovascular Trials Initiative (CDISC) draft “Standardized Definitions for Cardiovascular and Stroke Endpoint Events in Clinical Trials”. The definition of myocardial infarction complies with the “2012 Third Universal Definition of Myocardial infarction”.

Except for death and revascularisation, outcome events were identified based on a review of medical discharge records and registered discharge diagnoses. Consequently, potential events of myocardial infarction, stroke, or TIA required the patient to be admitted to hospital and hospitalised to be included as outcome events. Identification of revascularisation events was based on registry data, which included both in-patient and out-patient procedures performed at Östersund Hospital or the University Hospital of Northern Sweden. The patient medical record is electronic and connected with the National Civil Register. When a patient died within the national boarders of Sweden, this information, including the date of death, was usually available within 24 hours. All care givers within Jämtland-Härjedalen used the same electronic medical record. Thus, for events of death occurring within the county, all medical documentation except for forensic protocols was available to the reviewers. In a few cases (n=14), the underlying cause of death could not be classified as cardiovascular or non-cardiovascular due to absent or insufficient documentation.

**Outcome events**

1. **Death**

Classified as cardiovascular, non-cardiovascular, or undetermined.

1. **Cardiovascular death**

Includes death due to any of the following conditions:

1. Acute myocardial infarction

As defined below or verified by autopsy. Includes death by any cardiovascular mechanism (arrhythmia, sudden cardiac death, congestive heart failure, stroke, pulmonary embolism, peripheral artery disease, invasive revascularisation procedure) within 30 days of an acute myocardial infarction.

1. Sudden cardiac death

Unexpected death, *not* caused by acute myocardial infarction. Includes death according to any of the following scenarios:

1. Witnessed and occurring without new or worsening symptoms;
2. Witnessed within 60 minutes after onset or worsening of cardiac symptoms;
3. Witnessed or unwitnessed with arrhythmia identified by ECG recording, defibrillator monitoring, or implantable device such as cardioverter defibrillator or loop recorder;
4. Death after unsuccessful resuscitation from cardiac arrest;
5. Death after successful resuscitation from cardiac arrest without identification of a specific cardiac or non-cardiac aetiology;
6. Patients found dead within 24 hours of last being seen well and stable and without signs of a specific non-cardiovascular cause.

1. Congestive heart failure

Death in association with clinical worsening of symptoms of congestive heart failure regardless of aetiology (ischaemic heart disease, non-ischaemic cardiomyopathy, valvular disease).

1. Stroke

Death as a direct consequence of stroke or indirectly due to a related complication.

1. Cardiovascular procedure

Death caused by immediate complication of a cardiac procedure.

1. Cardiovascular bleeding

Death due to any of the following:

1. Non-stroke intracerebral haemorrhage;
2. Non-traumatic, non-procedural vascular rupture (e.g., aortic aneurysm);
3. Cardiac tamponade.
4. Other

Death due to other cardiovascular conditions, such as pulmonary embolism or peripheral artery disease.

1. **Non-cardiovascular death**

Documentation supporting death due to a specific, non-cardiovascular cause, such as:

1. Pulmonary;
2. Renal;
3. Gastrointestinal;
4. Pancreatic;
5. Infection (includes sepsis);
6. Suicide;
7. Trauma;
8. Malignancy.
9. **Undetermined cause of death**

Death cannot be classified as cardiovascular or non-cardiovascular due to absent documentation.

1. **Acute myocardial infarction**

Based on 2012 Third Universal Definition of Myocardial Infarction. Acute myocardial infarction should be used when there is evidence of myocardial necrosis in the clinical setting of myocardial ischaemia, i.e., any of the following should apply:

1. Rise and/or fall of cardiac biomarkers with at least one measurement above the 99^th^ percentile of the upper reference limit (URL) in combination with at least one of the following:
   1. Ischaemic symptoms;
   2. New or presumed new ECG changes (described in further detail below): ST-elevation, ST-depression, T-wave inversion, or LBBB;
   3. Development of pathological Q-wave (described in further detail below);
   4. Evidence by imaging of loss of viable myocardium or new regional wall motion abnormality;
   5. Intracoronary thrombus identified by coronary angiography or autopsy.
2. Sudden cardiac death preceded by clinical symptoms of cardiac ischaemia *and* new onset ischaemic ECG changes in a patient in whom death occurred before cardiac biomarkers were obtained or would be increased.
3. Percutaneous coronary intervention (PCI)-related MI: elevation of cardiac biomarkers >5 times the URL (or increased >20% in a patient with stable elevation or falling values) *and* at least one of the following:
   1. Ischaemic symptoms;
   2. Ischaemic ECG changes;
   3. Angiographic evidence of procedural complication;
   4. Evidence by imaging of loss of viable myocardium or new regional wall motion abnormality.
4. Stent thrombosis detected by coronary angiography or autopsy in combination with ischaemic symptoms and a rise/fall of cardiac biomarkers with at least one value above the 99^th^ percentile of the URL.
5. Coronary artery bypass grafting (CABG)-related MI: elevation of cardiac biomarkers (>10 times the URL) in a patient with normal baseline levels in combination with any of the following:
   1. New Q-wave or LBBB;
   2. Angiographic evidence of occlusion of a new graft or a native coronary artery;
   3. Evidence by imaging of loss of viable myocardium or new regional wall motion abnormality.

**Subclassification of myocardial infarction:**

**Type 1:** Spontaneous myocardial infarction

Plaque rupture, ulceration, fissuring, erosion or dissection resulting in intraluminal thrombus and decreased myocardial blood flow.

**Type 2:** Myocardial infarction secondary to an ischaemic imbalance

A condition other than coronary artery disease contributes to an imbalance between myocardial oxygen demand and supply, resulting in myocardial necrosis.

**Type 3:** Myocardial infarction resulting in death when biomarker values are unavailable

**Type 4a:** Myocardial infarction related to PCI

**Type 4b:** Myocardial infarction related to stent thrombosis

**Type 5:** Myocardial infarction related to CABG

**Criteria for ECG changes suggestive of ischaemia:**

**STEMI:** *ST-elevation* in two contiguous leads with cut-points: 1 mV in all leads other than lead V2-V3 where the following cut points apply: 2 mV for men ≥40 years; 2·5 mV for men <40 years; 1·5 mm for women.

**NSTEMI:** *ST-depression* ≥0·05 mV in two contiguous leads or *T-wave inversion* ≥0·3 mV in two contiguous leads with prominent R-wave or R/S ratio>1.

**Prior myocardial infarction:** *Q-wave* in V2-V3 ≥0·02 sec or QS complex in V2-V3. Q-wave ≥0·03 sec and ≥1 mV deep in I, II, aVL, aF, or V4-V6 in any two leads of a contiguous lead grouping.

1. **Revascularisation**

Invasive revascularisation regardless of indication.

1. PCI with balloon or stent;
2. CABG.
3. **TIA and stroke**

The distinction between a TIA and an ischaemic stroke is the presence of infarction. Duration of symptoms for 24 h is interpreted as presence of infarction, even when evidence is absent on imaging.

1. TIA

Transient ischaemic attack is defined as a transient (within 24 h) episode of focal neurological dysfunction caused by brain, spinal cord, or retinal ischaemia without acute infarction.

1. Ischaemic stroke

Ischaemic stroke is defined as an acute episode of focal cerebral, spinal, or retinal dysfunction caused by infarction of the central neurons system.

1. Haemorrhagic stroke

Acute episode of focal or global cerebral or spinal dysfunction caused by spontaneous intracerebral haemorrhage (excluding subarachnoid haemorrhage).

1. Undetermined

Acute episode of focal or global cerebral, spinal, or retinal dysfunction caused by presumed infarction or haemorrhage, but with insufficient information to allow categorisation (i.e., imaging not performed).
