# Supplementary material for: Smoking cessation and prognosis during long-term follow-up after stroke, TIA, and acute coronary syndrome—results from the randomized controlled NAILED trial
Source: PLoS One. 2024 Nov 11;19(11):e0311955. doi: 10.1371/journal.pone.0311955 (PMC11554128; doi:10.1371/journal.pone.0311955)
Supplement: Attachment — Submitted filename: Response_to_reviewers.docx [file pone.0311955.s005.docx]

Dear Editor and Reviewers,

Thank you for your time and for valuable comments on the manuscript. Please find our point-by-point response below. Reviewer #2 expressed concern regarding one of the secondary outcomes: The association between early smoking cessation and prognosis. After careful consideration, including the comments by reviewer #1, we have chosen to keep this outcome in the manuscript. The outcome of concern is presented as exploratory and we believe that it serves a value in describing the high risk associated with continued smoking, which is of importance to motivate further studies of interventions to improve early smoking cessation rates. If the Editor does not share our opinion, we are, of course, open to further discussion including suggestions of improvements.

The resubmitted version of the manuscript is revised to adhere to the journal style requirements and a statement regarding trial registration has been added. All changes to the manuscript are marked in the manuscript with tracked changes file.

Best Regards,

Anna-Lotta Irewall, on behalf of all the authors

**Point-by-point response**

**Journal requirements:**

1. We have now revised the manuscript, figures and tables to adhere to the PLOS ONE style requirements as specified in the templates. Please let us know if there are any remaining formatting issues that we have not addressed.

2. We have now added information regarding trial registration to the title page. Please note that the NAILED trial appears in the ISRCTN registry under three different registration numbers. This is further explained under the trial registration headline in the methods section (page 10, lines 221-228). In accordance with the comment above we have added a statement confirming that all ongoing and related trials are registered (page 10, lines 228-229). The time period for participant recruitment and follow-up is found in the methods section (page 6, lines 111-112, and page 9, line 183).

3. Data requests may be sent to Umeå University through the e-mail address [registrator@umu.se](mailto:registrator@umu.se). This address is given in the submission form. In Sweden the universities are legally obligated to store research data for a minimum of 10 years to ensure future availability.

We have reviewed reference list and it should be correct.

**Review comments**

**Reviewer #1 comments:**

*“A very well-written article as part of the NAILED trial. The study showed that smoking cessation advise by a nurse had no effect on smoking cessation after a cardiovascular event. Moreover, the likelihood of quitting smoking would be early after a CV event and unlikely to do so in long-term followup.

It would be interesting to look into the amount of cigarettes and duration of smoking prior to the CV event and compare those who quit with those who did not as perhaps those who smoke longer and higher amounts may have more difficulty in quitting. However as this study is a subanalysis it may be difficult to determine.

Overall this paper is good as it would change the practice and explore other ways for patients to quit smoking after a CV event.”*

Response:

Thank you for your positive review. We agree that it would indeed be interesting to look into patterns related to the number of cigarettes smoked and duration of smoking. Smoking “dose” and duration of smoking would have been of interest both for the primary and for the secondary, explorative, outcome. It would also have been relevant to assess if the intervention had any effect on the number of cigarettes smoked among those who did not quit. Unfortunately, data collection regarding smoking was limited to smoking status at baseline (current/former/never) and each follow-up occasion (yes/no). We were therefore unable to perform any of those analyses. This is a weakness of the study which is mentioned in the last part of the discussion (page 24, line 502-504).

**Reviewer #2 comments:**

*“This is a substudy of NAILED series RCTs. And the authors want to show the nurse-led telephone intervention on the smoking cessation and the consequent results. The primary endpoint is a negative result.
1. It is very confused of the setting of clinical endpoint. The intervention method seems to be nurse-led telephone follow-up, and what happened to this intervention should be focused. The primary endpoint is OK. But the secondary endpoint was the association between early smoking cessation and prognosis, which is a little far away from the design.
2. The study is less of novelty. Although the secondary endpoint is positive, it dose not give novel knowledge.”*

Response:

We do agree that the intervention effect on smoking cessation should be the main focus of the manuscript as that was the aim of this randomized controlled sub study. In our opinion, that is well reflected by the primary outcome and also by secondary outcomes with focus on between-group differences in smoking status at different time-points during follow-up. Correspondingly, these outcomes are also the main focus of the results and the discussion. In addition, we chose to include the association between smoking cessation and prognosis as an explorative, secondary outcome because assessment of cardiovascular risk and the effect of smoking cessation is of importance to motivate interventions to improve smoking cessation rates. Brief counseling is probably the most common intervention used to support smoking cessation in clinical practice and sometimes also included in guidelines despite little evidence for the effectiveness of such interventions among patients with cardiovascular disease. In this context, the neutral, main result of our study makes an important contribution and also the pattern of smoking cessation during long-term follow-up provide novelty. When it comes to the exploratory secondary outcome, we do agree that this result represents less of novelty as corresponding associations have been previously shown. However, reproduction of results in different populations does still hold a value and a major strength of this study is the populations’ high resemblance with the target population found in clinical practice.
