# Supplementary material for: Smoking cessation and prognosis during long-term follow-up after stroke, TIA, and acute coronary syndrome—results from the randomized controlled NAILED trial
Source: PLoS One. 2024 Nov 11;19(11):e0311955. doi: 10.1371/journal.pone.0311955 (PMC11554128; doi:10.1371/journal.pone.0311955)
Supplement: Attachment — Submitted filename: Response_to_reviewers_R2.docx [file pone.0311955.s006.docx]

Dear Editor and Reviewer,

Thank you for your time and for valuable comments on the manuscript. We have now carefully considered all comments in consultation with our statistician and several changes to the previous version of the manuscript have been done in order to address the issues pointed out by the statistical reviewer. As requested by the editor, we have also uploaded the figure files to PACE and as far as we can tell they seem be of adequate quality. We have also reviewed the reference list again and replaced two references. Please find our point-by-point response below. All changes to the manuscript are marked in the manuscript with tracked changes file (except for changes to the reference list which are highlighted in yellow).

Best Regards,

Anna-Lotta Irewall, on behalf of all the authors

**Point-by-point response**

**Journal requirements:**

*“Please review your reference list to ensure that it is complete and correct. If you have cited papers that have been retracted, please include the rationale for doing so in the manuscript text, or remove these references and replace them with relevant current references. Any changes to the reference list should be mentioned in the rebuttal letter that accompanies your revised manuscript. If you need to cite a retracted article, indicate the article’s retracted status in the References list and also include a citation and full reference for the retraction notice.”*

In this second review of the reference list, we noticed that the article by Critchley et al had been retracted. In addition, we identified a very recently published update of the review by Rigotti et al. We have now replaced the retracted article by a more recently published Cochrane review by Wu et al (ref 5) and the review by Rigotti et al has been replaced by the update by Streck et al (ref 24). These changes to the reference list could not be done while using the “tracked changes tool”. Instead we have manually highlighted the new references in yellow. Please let us know if you find the update unclear in any way.

**Review comments**

**Reviewer #3 comments:**

*“Flowchart: If smoking status is checked after randomization, it is very unlikely to have same numbers of smokers the same between the intervention and control groups. There was no loss to follow-up over 8 years?”*

Baseline characteristics including smoking status were collected in-hospital for all identified patients prior to randomization. To clarify this, we have revised the first sentences under the sub heading *Data collection* in the methods section (pages 8, lines 169-172). With a sample of the current size (n=1890) the resulting groups are expected to end up well balanced in terms of baseline characteristics, all though some differences can still occur by chance. To minimize the risk of unbalance in certain key variables that could severely compromise the internal validity of the study, the randomization was stratified (further discussed below). The randomization was, however, *not* stratified for smoking status. Luckily for the current sub study, this variable did not deviate from the expected equal distribution between the randomized groups.

Thank you for your valuable comment on the flowchart. Most participants did not take active part in the study assessments for 8 years. Depending on the year of inclusion, the maximum duration of follow-up was 3 to 8 years (described on page 8, lines 173-174, and in the figure legend). There was also participants that withdrew during the course of the study and participants in which follow-up was discontinued due to death. In response to your comment, we have now revised the flowchart (figure 1) to clarify discontinuation due to withdrawal or death.

*“The stratification is unclear regarding the use of "either one factor or another". Is the randomization stratified by both factors? The stratification factor (e.g., Rankin scale) should be included in Table 1. Stratification factors need to show balanced in Table 1.”*

Thank you for pointing out ambiguity in the sentence describing the randomization process (page 6, lines 121-124). We have now rephrased the sentence by exchanging the word “subgroup” for “qualifying event”. We hope that you will find this change as clarifying. As requested, we have also added modified Rankin Scale as a variable in table 1.

*“Power Calculation: Clarify that a chi-square test was used. Justify the effect size of a 20% difference. Additionally, if the sample size was not determined by power calculation during the study design stage, the post hoc power calculation is not meaningful.”*

We believe that an effect size of 20% is what could be considered a clinically relevant difference for this kind of intervention and we included a post-hoc power calculation to show that our study sample was large enough to detect such an effect with reasonable power. We do, however, acknowledge that the value of post hoc power calculations is a matter of debate and, after consideration, we have now removed this calculation from the manuscript as a response to your comment.

*“If an intent-to-treat analysis was used, the analyzed sample size should be the same as the randomized number.*”

The comparison of the proportion of non-smokers at 1 and 3 years did not include participants that had died by these respective time points, which we find reasonable. With that exception, the analyses did comprise all randomized participants included in this sub study (n=321). Death or withdrawal before the first study follow-up (1 month post discharge) was an exclusion criteria of this sub study and that could, perhaps, be seen as a violation to the intention-to-treat principle. However, the construction of the outcomes required smoking status to be registered at least once after hospital discharge and for that reason participants that did not remain in that study at 1 month could not be included in any analyses. As shown in figure 1, deaths and withdrawals between hospital discharge and the 1 month follow-up were well balanced between the study groups (n=11).

*“What model was used to handle competing risks?”*

The competing risk analysis was performed according to Fine-Gray. This information has now been added to the statistical methods section (page 10, line 214).

*“For secondary endpoints, p-values should be adjusted for multiple comparisons, e.g., the p-value for comparisons at 3 months.”*

We acknowledge the risk of type I error associated with multiple comparisons. However, several of the secondary outcomes were related (smoking status at one time point was highly predictive of smoking status at a later time point) and for that reason we believe that correction for multiple comparisons would have been too conservative in this study. Also, adjustment for multiple comparisons was not specified in the study protocol. After consideration, we have therefor chosen to keep the analyses for secondary outcomes without adjustment. Instead, we have now added the issue with multiple comparisons as a weakness in the discussion (page 24, line 491-492).
